# Recognition and Localization of Maize Leaf and Stalk Trajectories in RGB Images Based on Point-Line Net

**DOI:** 10.34133/plantphenomics.0199

**Published:** 2024-07-09

**Authors:** Bingwen Liu, Jianye Chang, Dengfeng Hou, Yuchen Pan, Dengao Li, Jue Ruan

**Affiliations:** ^1^ College of Computer Science and Technology (College of Data Science), Taiyuan University of Technology, Taiyuan 030024, Shanxi, China.; ^2^ Shenzhen Branch, Guangdong Laboratory for Lingnan Modern Agriculture, Genome Analysis Laboratory of the Ministry of Agriculture and Rural Affairs, Agricultural Genomics Institute at Shenzhen, Chinese Academy of Agricultural Sciences, 518120 Shenzhen, China.

## Abstract

Plant phenotype detection plays a crucial role in understanding and studying plant biology, agriculture, and ecology. It involves the quantification and analysis of various physical traits and characteristics of plants, such as plant height, leaf shape, angle, number, and growth trajectory. By accurately detecting and measuring these phenotypic traits, researchers can gain insights into plant growth, development, stress tolerance, and the influence of environmental factors, which has important implications for crop breeding. Among these phenotypic characteristics, the number of leaves and growth trajectory of the plant are most accessible. Nonetheless, obtaining these phenotypes is labor intensive and financially demanding. With the rapid development of computer vision technology and artificial intelligence, using maize field images to fully analyze plant-related information can greatly eliminate repetitive labor and enhance the efficiency of plant breeding. However, it is still difficult to apply deep learning methods in field environments to determine the number and growth trajectory of leaves and stalks due to the complex backgrounds and serious occlusion problems of crops in field environments. To preliminarily explore the application of deep learning technology to the acquisition of the number of leaves and stalks and the tracking of growth trajectories in field agriculture, in this study, we developed a deep learning method called Point-Line Net, which is based on the Mask R-CNN framework, to automatically recognize maize field RGB images and determine the number and growth trajectory of leaves and stalks. The experimental results demonstrate that the object detection accuracy (mAP50) of our Point-Line Net can reach 81.5%. Moreover, to describe the position and growth of leaves and stalks, we introduced a new lightweight “keypoint” detection branch that achieved a magnitude of 33.5 using our custom distance verification index. Overall, these findings provide valuable insights for future field plant phenotype detection, particularly for datasets with dot and line annotations.

## Introduction

Maize, as an essential cash and food crop worldwide, not only provides a significant amount of food and feed for humans but also has extensive applications in industry, bioenergy, and other sectors [1–3]. The examination of maize phenotypes—external morphological and physiological traits such as plant height [4,5], leaf number [6–8], and leaf length [9]—is essential for increasing yield and precise breeding. For instance, timely access to crop leaf counts can guide farmers to take precise management measures, including the use of appropriate fertilization, irrigation, and working conditions. In addition, the structure of plants can be obtained over time through trajectory capture, providing valuable data to agricultural researchers to help them better understand the causes and mechanisms of plant type formation to achieve accelerated and targeted breeding. Nevertheless, obtaining the phenotypic characteristics of crops in the field is a challenge because accurate statistical methods are required to reduce errors, and the statistics of crop morphological structure itself have a certain degree of subjectivity. Consequently, automated, objective, and reliable techniques are indispensable for the detection of maize field phenotypes to enhance efficiency and reduce resource consumption in maize phenotype research.

In recent years, the field of plant phenotype detection has undergone notable advancements due to the rapid development of computer vision technology, especially deep learning [10,11]. Convolutional neural networks (CNNs) have achieved substantial success in object classification [12–15], detection [16–19], and segmentation [20–23]; have been applied on a large scale in different fields; have greatly boosted productivity in these areas; and have achieved considerable economic benefits [24]. The keypoint detection task involves using CNNs or other deep learning models to accurately pinpoint the location of structurally or semantically important points, such as human body joints, object corners, or salient features, within an image, which results in a large splash [25]. The application of keypoint detection methods in the field of plant phenotype detection has great potential for providing new ideas and methods for accurate monitoring and analysis of plant growth status. For example, keypoint detection algorithms are used to identify and localize key feature points in plant structures, such as the apex of leaves and the connection points of leaf veins. Accurate detection of these keypoints can help researchers understand the structural composition, growth and development process, and environmental adaptability of plants, which, in turn, can provide important references and support for breeding selection, disease diagnosis, and agricultural production. The in-depth study of the keypoint detection task for plant phenotypes will help to promote the development of agricultural production and botanical research. Therefore, it is very valuable to explore the powerful capabilities of CNN-based keypoint detection methods in maize phenotypic image processing and understanding. However, relatively little research has been conducted in the field of plant phenotype detection.

In the field of phenotypic detection in maize, using CNNs, Zhou et al. developed Maize-IAS, a software that can perform functions such as counting maize leaves indoors, facilitating agricultural research [26]. Based on the Faster R-CNN training model, Miao et al. [27] indirectly calculated the number of maize leaves by predicting the number of indoor maize and sorghum leaf tips, which improved the accuracy of crop monitoring. In addition, Ao et al. [28] developed a 2-stage approach based on LiDAR data that combines CNNS and morphological features to segment the stems and leaves of individual corn plants in a field setting, which offers a new solution for precision agriculture. Xu et al. [29] used RGB images collected by an unmanned aerial vehicle (UAV) to construct a 2-stage model and detected and counted maize seedling leaves. This method can quickly assess the growth of crops, which is highly important for agricultural production.

Although many deep learning models have been developed to detect specific phenotypes of corn, most of these models are conducted indoors or in simple controlled environments, and they often rely on expensive data, such as radar point clouds, or are based on deep learning techniques. However, these models are not specifically designed for leaf identification and tracking capture. In the field, the detection of corn stems and leaves faces many challenges, mainly because the following factors are more complex than in the indoor environment: background diversity, severe shading between leaves, unpredictable weather conditions, and changing light conditions, which can greatly affect the performance of the detection algorithm. Here, inspired by the keypoint detection strategy, we create a field corn detection model named Point-Line Net based on a special RGB dataset [30] to accurately locate leaf positions and track leaf trajectories in a field environment. We believe that the results of this study can also provide ideas for field management and phenotypic data collection for other crops.

## Materials and Methods

### Maize dataset

**Fig. 1.**
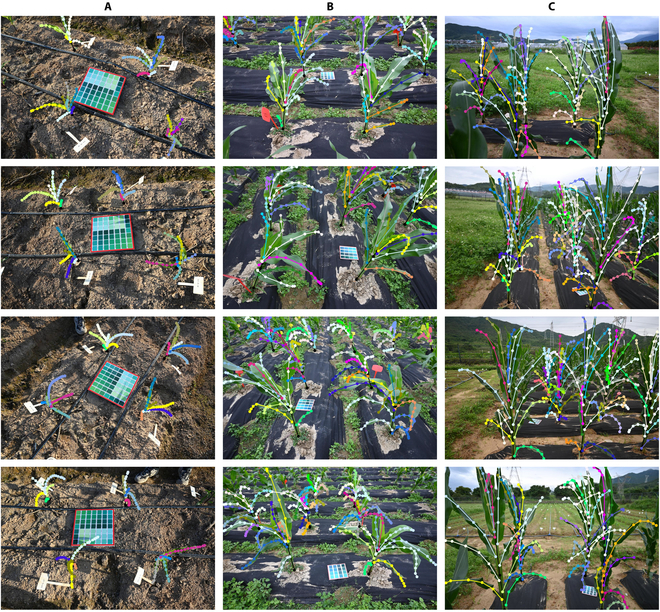
Images at 4 angles of the maize dataset with annotations for 3 growth periods: (A) early stage, (B) middle stage, and (C) late stage.

The maize dataset used in this study, as shown in Fig. 1, was provided by the Agricultural Genomics Institute, Chinese Academy of Agriculture Sciences (CAAS) and is now included in the Maize Image Phenotyping Database (MIPDB) database [30]. Our team constructed a comprehensive maize image phenotypic dataset, which was collected using handheld cameras and UAVs at multiple angles and time intervals. The dataset encompasses more than 30,000 high-resolution RGB images, of which more than 17,000 leaves and stalks were manually labeled using Labelme [31] software, and all the maize images were divided into 3 distinct periods based on growth cycles. For this study, due to noteworthy disparities in morphology and background between maize images captured by cameras and those obtained via UAVs, we decided to exclusively analyze and process the maize images acquired through cameras to attain superior results. Nevertheless, there is immense potential to extract additional insights from multiple-angle and multiple-time series data to make further progress, which we will discuss in Discussion.

Compared to the currently widely used plant phenotypic datasets, which are primarily annotated with segmentation masks or target detection rectangles [32–34], our maize dataset takes a novel approach by utilizing dotted-line annotation to depict leaf and stalk trajectories, as illustrated in Fig. 1. The method offers the advantage of conveying a more precise representation of these trajectories. When target positions and contours are labeled using segmentation masks, semantic segmentation or instance segmentation algorithms [35] often generate extraneous pixels. However, not all pixels are required to describe leaf and stalk trajectories. In addition, it is inappropriate to use only rectangular bounding boxes to annotate target positions, as unlike in the laboratory [32], there are significant occlusion issues with maize leaves under field conditions, especially in later stages. This poses an important challenge that we need to address in our work. Hence, the use of dotted line annotation is more suitable for field-based maize phenotype detection and provides researchers with greater challenges.

### Dataset processing

The original field images in our dataset have a size of 6,016 × 4,016. Considering the challenges associated with memory management on available Graphics Processing Units (GPUs), we manually downsampled the original images by a factor of 4 to 1,504 × 1,004 for training and validation. We also downscaled the coordinate information stored in the corresponding annotation file accordingly. This downsampling approach was adopted to address the limitations of memory size while aiming to retain the RGB information as much as possible.

**Fig. 2.**
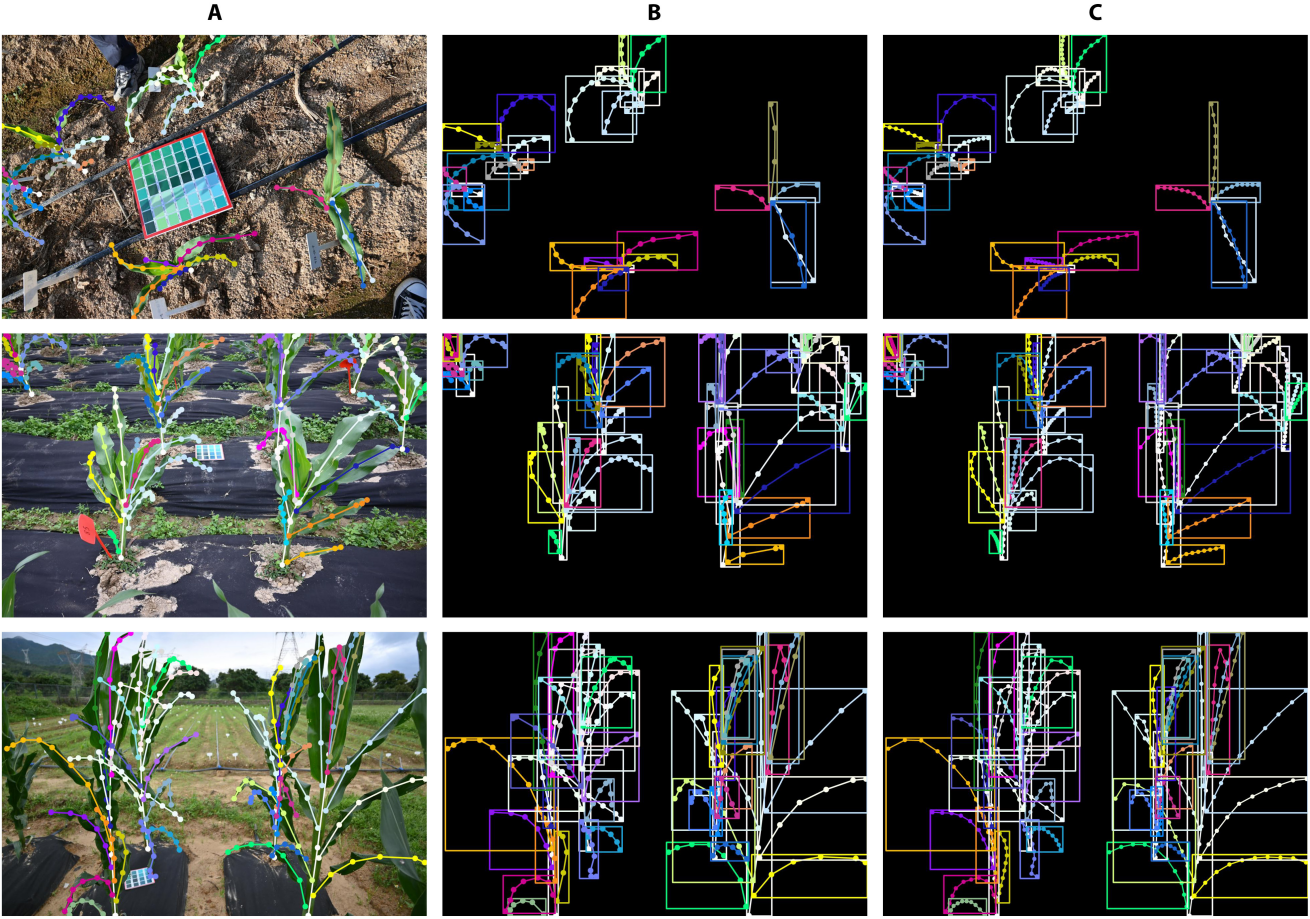
Method of determining the ground-truth bounding box. (A) Original annotation. (B) Transformed bounding box annotation. (C) Keypoint annotation after interpolation algorithm processing.

The annotation file is formatted as a JSON file containing comprehensive annotation information, including the target category (such as leaf or stalk), the serial number, and the coordinate information of the annotated points. As mentioned previously, our original dataset does not provide rectangular bounding boxes for target detection considering the desire to solve the target occlusion problem. However, our proposed approach based on Mask R-CNN requires the annotation of actual target detection boxes [36]. To address this, we calculate the coordinates of the points marked for each individual target, determining the minimum coordinate values as the upper-left point of the target detection box and the maximum coordinate values as the lower-right point of the target detection box. We also added a certain offset (20) for each calculated coordinate point to include the full target, as depicted in Fig. 2. In this way, the generated rectangular box serves as the ground truth for the training process.

Additionally, inspired by the multiperson human keypoint detection task [37,38], our proposed approach aims to extend the object detection task to a keypoint detection task, and further details will be discussed in the “Modification of the keypoint detection branch” section. To make cluttered raw keypoint annotations more learnable, we uniformly process the dataset using the following method. First, we employ an interpolation algorithm to expand the initially small number of labeled keypoints. Then, for each ground-truth object, we traverse the keypoints contained within it and extract a portion of them as ground-truth keypoints at equal intervals based on a predetermined ratio (30%). The final data processing results are shown in Fig. 2. Finally, the preprocessed dataset is randomly partitioned at a ratio of 7:2:1 to obtain the training, validation, and test sets for subsequent model training and performance evaluation.

### Maize detection

#### Point-Line Net-based Mask R-CNN

Considering the distinctive dotted-line annotation in our dataset, we draw a parallel between this annotation and the task of human pose estimation [38]. Human pose estimation, often referred to as keypoint detection, is a computer vision task that involves locating and identifying specific points on a person’s body, such as joints, limbs, and facial features. The objective of keypoint detection is to accurately estimate the 2D or 3D positions of these points in an image or video. Similar to the instance segmentation task, keypoint detection can be categorized into 2 approaches—top-down and bottom-up—based on pose formation logic. The top-down approach, which relies on object detection [36], involves first detecting the regions in the image where each instance is presented and then performing a separate semantic segmentation task or keypoint detection on these candidate regions. This approach is constrained by the accuracy of the object detection stage. On the other hand, the bottom-up approach [35] treats instance segmentation or multiperson keypoint detection as a clustering task, grouping pixels or global keypoints into an arbitrary number of target instances within the image. The class of each group is then determined to achieve instance segmentation or keypoint detection. Given the complex background of the field and the challenge of overlapping shading, particularly in later stages, we propose an improved top-down approach based on Mask R-CNN [36] named Point-Line Net (https://github.com/VEGETALOADING/Point-Line-Net), which aims to address these challenges and improve the accuracy of keypoint detection in our dataset.

**Fig. 3.**
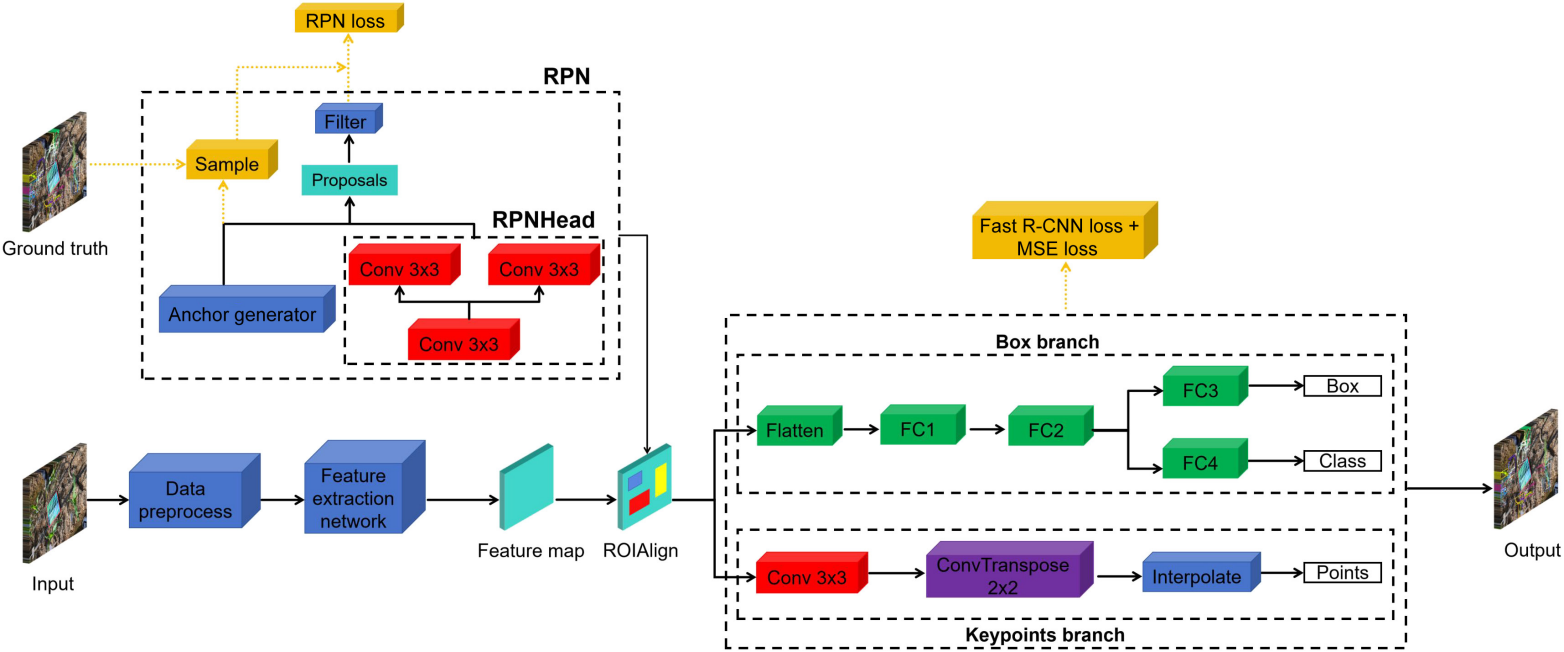
Point-Line Net structure based on the Mask R-CNN model: in the box branch, the model inference focuses on the bounding boxes of targets as well as their category, and in the keypoint branch, the model inference focuses on the heatmap of the keypoints in conjunction with the results of the object detection.

Mask R-CNN is an extension of Faster R-CNN [39], as depicted in Fig. 3. Faster R-CNN consists of 2 main components: a region proposal network (RPN) and a Fast R-CNN detector. The RPN generates a set of object proposals, which are then input into the Fast R-CNN detector for object classification and bounding box regression. The RPN is trained to learn objectness scores and bounding box regressions, while the Fast R-CNN detector is trained to classify the objects within each proposal and refine the bounding boxes. Building upon Faster R-CNN, Mask R-CNN extends the framework by predicting a binary mask or a set of heatmaps for keypoints for each detected object. The mask indicates the pixelwise location of the object within the image, while the heatmaps indicate the specific locations of keypoints on the object within the image.

Traditional ideas for keypoint detection can be categorized into regression-based and heatmap-based approaches. Regression-based keypoint detection methods are implemented by directly predicting the coordinate values of target keypoints [38]. Typically, the method utilizes a CNN regressor to learn the mapping from the input image to the target keypoint coordinates. In the training phase, the input image and its corresponding target keypoint coordinates are required as training samples, and then the parameters of the regressor are optimized by minimizing the error between the predicted keypoint coordinates and the actual coordinates.

Despite the overall simplicity and efficiency of this approach, large-scale training data and complex network structures are required to learn effective mapping relationships between feature representations and keypoint locations due to the inherent limitations of CNNs in regressing long-distance offsets. As a result, such methods often cannot achieve the same level of accuracy as heatmap-based methods.

Heatmap-based methods [40] do this by predicting the heatmap of the probability distribution of each pixel point belonging to each keypoint. In the training phase, a heatmap conforming to a Gaussian distribution is usually generated as the target label with the target keypoint as the center coordinate, and finally, the model is trained by minimizing the difference between the ground-truth heatmap and the predicted heatmap.

Although the heatmap-based approach requires greater computational and storage overhead and additional postprocessing steps such as nonmaximum suppression (NMS) to extract the final keypoints, this approach is easier to optimize because it does not require direct regression of the keypoint coordinates but rather predicts the probability distribution of pixels belonging to each keypoint. In addition, since each pixel point can predict the location of multiple keypoints, the case of mutual occlusion between target keypoints can be better handled.

Unlike Mask R-CNN, which incorporates a third branch for traditional heatmap-based keypoint detection into the network, our approach modifies Faster R-CNN by introducing a branch to predict “keypoints” for each detected object using a single heatmap, as illustrated in Fig. 3, instead of using heatmaps for every keypoint. Additionally, the proposals generated by the RPN are also utilized in the keypoint head for keypoint detection. Further details of the keypoint detection branch are presented in the “Modification of the keypoint detection branch” section.

#### Adjustment of hyperparameters

We adjusted several hyperparameters and conducted corresponding experiments. First, we increased the number of proposals retained in the RPN before and after NMS processing, namely, RPN PRE NMS and RPN POST NMS. The numbers increased from 2,000 to 4,000 and from 1,000 to 2,000, respectively. This modification aimed to retain more candidate proposals, considering that our dataset often features a larger number of targets, and the distance measured by intersection over union (IoU) between targets is closer. The results, as demonstrated in the “Object detection results for maize based on three different original models” section, indicate that this adjustment yields the best performance for object detection in our model.

Additionally, regarding the anchors, we expanded the number of scales to include 5 different box areas, namely, 32^2^, 64^2^, 128^2^, 256^2^, and 512^2^ pixels. This adjustment was experimentally proven to accelerate the convergence of the model and improve its performance, allowing it to adapt to targets of various scales. Furthermore, in the original Faster R-CNN [7], RoIPool is employed to align the proposals obtained from the RPN to the same size. However, this process involves rounding operations, resulting in less precise localization. To overcome this limitation, the authors proposed the RoIAlign method as an alternative to RoIPool, which provides more accurate spatial localization information. We have also incorporated this improvement into our model. Moreover, the size of the pooling operation not only impacts the expressiveness of the network but also affects the accuracy of the region of interest (RoI). If the pooling size is too small, such as 7, detailed information within the RoI may be lost, thereby hindering the detection results. Conversely, if the pooling size is too large, such as when the feature representation of the RoI becomes too coarse, the detection performance may degrade. Considering the characteristics of our dataset and the aforementioned analysis, we adjusted the output size of RoIAlign to 14, which yielded the optimal detection effect.

#### Soft-NMS

The NMS technique is commonly used in object detection algorithms to remove redundant and overlapping detections, resulting in a final set of bounding boxes with the highest confidence scores [41]. However, NMS has limitations. It may discard valid detections with lower scores if they overlap with a higher-score detection. Additionally, NMS selects only one detection for each object instance, which can lead to suboptimal performance when multiple detections are valid.

In the dataset used in this research, due to the large number of target instances that are close to each other or occluded from each other, the traditional NMS method may not be able to handle this complexity, which may lead to some correct targets being wrongly suppressed. To address these limitations, Soft-NMS [42] was introduced as a modified version of NMS that incorporates a soft weighting scheme. In Soft-NMS, each detection is assigned a weight based on its confidence score and the overlap it has with other detections. The weight of detection is determined by its score as well as the scores of all other detections that intersect with it. This weighting scheme allows for multiple detections of the same object instance to be retained while suppressing redundant detections. We believe that applying Soft-NMS to our maize dataset, which contains a significant amount of occlusion, can yield more accurate and robust results. Our experiments confirmed this hypothesis.

#### D IoU

In the target detection task, the IoU is used to perform NMS processing operations. The IoU measures the degree of overlap by calculating the ratio of the intersection area of 2 bounding boxes to their concatenation area. The specific calculation formula is as follows:

$$\mathrm{IoU}=\frac{A⋂B}{A⋃B}$$ (1)

where *A* and *B* represent the predicted and ground-truth bounding boxes, respectively. When the IoU ranges from 0 to 1, the larger the value is, the greater the degree of overlap of the 2 bounding boxes. When the IoU is equal to 1, the 2 bounding boxes overlap completely; when the IoU is equal to 0, the 2 bounding boxes do not overlap.

This traditional computation only considers the area ratio of the overlapping area between the detection frame and the real target bounding box without considering the distance information between them. When dealing with dense scenes such as those appearing in the dataset used in this research, the traditional IoU computation is prone to errors because the target instances may be occluded from each other or be in close proximity to each other, resulting in the incorrect prediction or omission of the bounding boxes of some real targets. Therefore, this method incorporates the distance-IoU (D IoU) [43] as an evaluation algorithm for filtering target bounding boxes to address the above problem.

D IoU [43] introduces the concept of considering the distance between the center points of bounding boxes to better measure the overlap between 2 closely positioned bounding boxes. In contrast, D IoU is more robust to variations in object sizes and aspect ratios. The traditional IoU may exhibit bias toward larger objects, whereas the D IoU can better handle scenarios where objects have different sizes and aspect ratios, as is often observed in our maize dataset. Therefore, the D IoU can provide a better reflection of the similarities and differences between 2 bounding boxes, avoiding the merging of bounding boxes that are in close proximity or overlapping. To improve the detection accuracy of closely positioned objects, we integrate the D IoU into the NMS process, which is represented as:

$$D_{\mathrm{IoU}}=\mathrm{IoU}-\frac{\rho^{2}\left(b,\;b^{\mathrm{gt}}\right)}{c^{2}}$$ (2)

where *b* denotes the centroid coordinates of the target bounding box for inference; *b^gt^* denotes the centroid coordinates of the ground-truth bounding box; *ρ*() represents the computation of the Euclidean distance; and *c* is the center point coordinate of the target bounding box that covers the inference as well as the center point coordinate of the diagonal length of the minimum rectangle of the ground-truth bounding box.

The calculation of D IoU introduces the penalty term of the distance from the center point of the bounding box, which can better distinguish between near and far targets, reduce the error due to the occlusion between targets or the proximity of the targets, and can effectively improve the accuracy of the target detector when dealing with dense target instances, which overcomes the limitations of the traditional IoU calculation method in this case. The algorithm integrates the D IoU computation strategy into the Soft-NMS algorithm, which makes the NMS family of algorithms more robust when dealing with the occlusion problem present in the dataset.

#### Modification of the keypoint detection branch

There are 2 main approaches to tackling the keypoint detection task: the regression-based approach and the heatmap-based approach. The regression-based approach directly estimates the keypoint coordinates (e.g., *x* and *y* coordinates) for each keypoint in the image. In this approach, the model is trained to predict the coordinates of each keypoint directly from the input image [44]. The output of the model is a set of coordinates that can be used to visualize the keypoints on the image. While this method is generally straightforward and efficient, it may not achieve the same level of precision as the heatmap-based approach due to the inherent limitations of CNNs in regressing long-distance offsets [45]. On the other hand, the heatmap-based approach [40] utilizes a deep neural network to analyze the image or video and generate a heatmap for each keypoint of interest. The heatmap indicates the likelihood of the keypoint being present at each location in the image or video. This approach is known for its higher accuracy but may involve more complex computations and slower processing compared to the regression-based approach.

The keypoint detection branch of Mask R-CNN is composed of fully connected layers that receive RoI-aligned features as input and generate a set of heatmaps, with each heatmap corresponding to a specific keypoint. However, as discussed in the “Maize dataset” section, the labeled points in our maize dataset that describe the position of the leaf or stalk are not specific. They are considered correct as long as they do not deviate from the leaf or stalk trajectories, which is different from the keypoints for the body used in human keypoint detection tasks such as OpenPose [46], where each of the 25 keypoints has precise location constraints. Based on the inference that these “keypoints” share similar characteristics in our dataset, we modified the traditional method of predicting keypoints. Instead of inferring a separate heatmap for each individual keypoint, we predict all keypoints within an object using a single heatmap. By doing so, we can obtain the positions of the peak values in the heatmap, which serve as the “keypoints” we need. This process is followed by applying NMS, as introduced in the “Soft-NMS” section, to determine the final locations of the keypoints.

#### Feature pyramid network

**Fig. 4.**
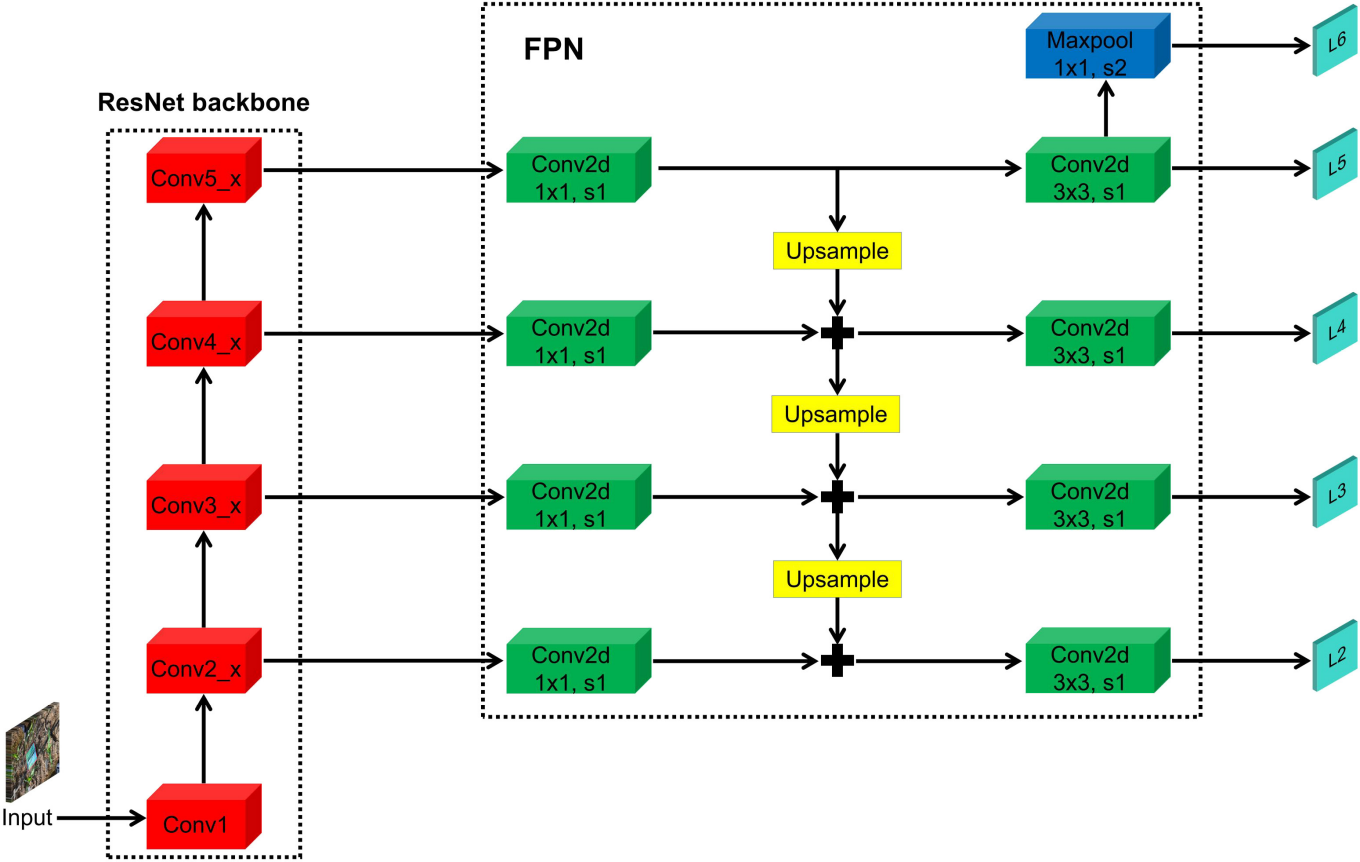
Feature pyramid network structure.

The feature pyramid network (FPN) [47] was proposed to address the challenge of detecting objects at different scales in an image, which is particularly relevant in our maize dataset where leaves exhibit significant variation in size and shape. FPN, as illustrated in Fig. 4, consists of a bottom-up pathway and a top-down pathway. The bottom-up pathway is a standard CNN that extracts features from the input feature maps. On the other hand, the top-down pathway is responsible for creating a feature pyramid, which is a set of feature maps at different scales based on the input. The top-down pathway takes the highest resolution feature map from the bottom-up pathway and upsamples it to the next scale. Subsequently, the upsampled feature map is merged with the feature map from the bottom-up pathway for that particular scale. This iterative process is repeated for each scale in the pyramid, resulting in a set of feature maps that are leveraged for object detection. FPN has been integrated with Mask R-CNN to further enhance object detection and segmentation performance. In the “Object detection results for maize based on three different original models” section, we will also present a comparison of our method to other approaches that do not employ FPN, where we demonstrate the superior performance of our proposed technique.

### Performance evaluation metrics

#### Mean average precision

The mean average precision (mAP) is a widely used metric for evaluating object detection algorithms. It serves as a measure of the accuracy and completeness of the detection results. The mAP is calculated by averaging the precision scores at various recall levels. Precision represents the ratio of true-positive detections to the total number of detections, while recall represents the ratio of true-positive detections to the total number of ground-truth objects. The formulas for precision and recall are as follows:

$$P=\frac{\mathrm{TP}}{\mathrm{TP}+\mathrm{FP}}$$ (3)

$$R=\frac{\mathrm{TP}}{\mathrm{TP}+\mathrm{FN}}$$ (4)

$$\mathrm{mAP}=\int_{0}^{1}P\left(R\right)\mathrm{dR}$$ (5)

In the first stage, Point-Line Net generates a list of bounding boxes along with their corresponding confidence scores. These confidence scores indicate the likelihood that the object inside the bounding box belongs to a particular class. Subsequently, the detection results are compared to the ground-truth objects, which consist of the actual objects in the dataset, including their respective bounding boxes and class labels. For each class, the precision and recall values are computed at various confidence thresholds. These values are then used to plot a precision–recall curve. The area under the curve (AUC) is calculated as a measure of performance. The mAP is obtained by averaging the AUC values across all classes. In our study, the mAP metric was evaluated based on IOU thresholds of 0.5 and 0.75, which are commonly employed for object detection tasks. A higher mAP indicates superior detection performance. The mAP metric is widely utilized in object detection benchmarks, including COCO [48], Pascal VOC [49], and ImageNet [50]. We also employ the mAP as a measure to assess the effectiveness of the object detection stage.

#### Mean line distance

In keypoint detection tasks, standard validation metrics typically rely on the object keypoint similarity (OKS) [51,52], which is represented as:

$$\mathrm{OKS}=\frac{\sum_{i}e^{-d_{i}^{2}/2s^{2}k_{i}^{2}}\xi \left(v_{i}>0\right)}{\sum_{i}\xi \left(v_{i}>0\right)}$$ (6)

where *d_i_* is the Euclidean distance between the detected keypoint and the corresponding ground truth, *v_i_* is the visibility of the ground truth, and *s* is the square root of the area size that is occupied by this object. However, such an evaluation metric, which is applicable to most keypoint detection tasks, is not suitable for our specific task. While our proposed method draws inspiration from keypoint detection, our goal is distinct. We aim to predict whether the keypoints are located on the “key path” (leaf or stalk trajectories) rather than requiring precise localization of each keypoint. Therefore, even if the predicted keypoints deviate slightly from the exact location, as long as they remain within the correct path, we consider this to be a correct prediction. Based on the aforementioned analysis, we developed a novel evaluation metric called the mean-line-distance (mLD) to assess the accuracy of keypoint detection for our specific requirements. The calculation process of the mLD is as follows:

$$C_{\left(x,y,x_{1},y_{1},x_{2},x_{2}\right)}=\left(x_{2}-x_{1}\right)*\left(x-x_{1}\right)+\left(y_{2}-y_{1}\right)*\left(y-y_{1}\right)$$ (7)

$$\begin{array}{c} L_{\left(x_{1},y_{1},x_{2},y_{2}\right)}=\\ \sqrt{\left(x_{2}-x_{1}\right)*\left(x_{2}-x_{1}\right)+\left(y_{2}-y_{1}\right)*\left(y_{2}-y_{1}\right)} \end{array}$$ (8)

Px,y,x1,y1,x2,y2=x1+x2−x1∗Cx,y,x1,y1,x2,x2Lx1,y1,x2,y2,y1+y2−y1∗Cx,y,x1,y1,x2,x2Lx1,y1,x2,y2 (9)

$$D_{\left(x,y,x_{1},y_{1},x_{2},y_{2}\right)}=\left\{\begin{array}{c} L_{\left(x,y,x_{1},y_{1}\right)},\;\text{if}\;C_{\left(x,y,x_{1},y_{1},x_{2},x_{2}\right)}\leq 0\\ L_{\left(x,y,x_{2},y_{2}\right)},\;\text{if}\;\frac{C_{\left(x,y,x_{1},y_{1},x_{2},x_{2}\right)}}{\sqrt{L_{\left(x_{1},y_{1},x_{2},y_{2}\right)}}}\geq 1\\ L_{\left(x,y,P_{\left(x,y,x_{1},y_{1},x_{2},y_{2}\right)}\right)},\;\text{otherwise} \end{array}\right.$$ (10)

$${\mathrm{LD}}_{k}=\frac{\sum_{i=0}^{M}\min\left(D_{\left(x_{i},y_{i},x_{g},y_{g},x_{g+1},y_{g+1}\right)},\;g\in G_{k}-1\right)}{M_{k}}$$ (11)

$$\mathrm{mLD}=\frac{\sum_{k=0}^{K}{\mathrm{LD}}_{k}}{K}$$ (12)

where *K* denotes the number of detected targets, *M* represents the number of keypoints that constitute each target after the postprocessing operation, and *G* refers to the number of keypoints of the ground truth that corresponded to each target. Consequently, *LD_k_* signifies the distance between the *k_th_* target and its corresponding real target, which is obtained by calculating the average distance between the *M* predicted keypoint and the real target. Given that each ground truth is labeled by multiple keypoints, we define the distance from the predicted point to the target as the shortest distance from the predicted point to the line segment formed by these multiple keypoints.

**Fig. 5.**
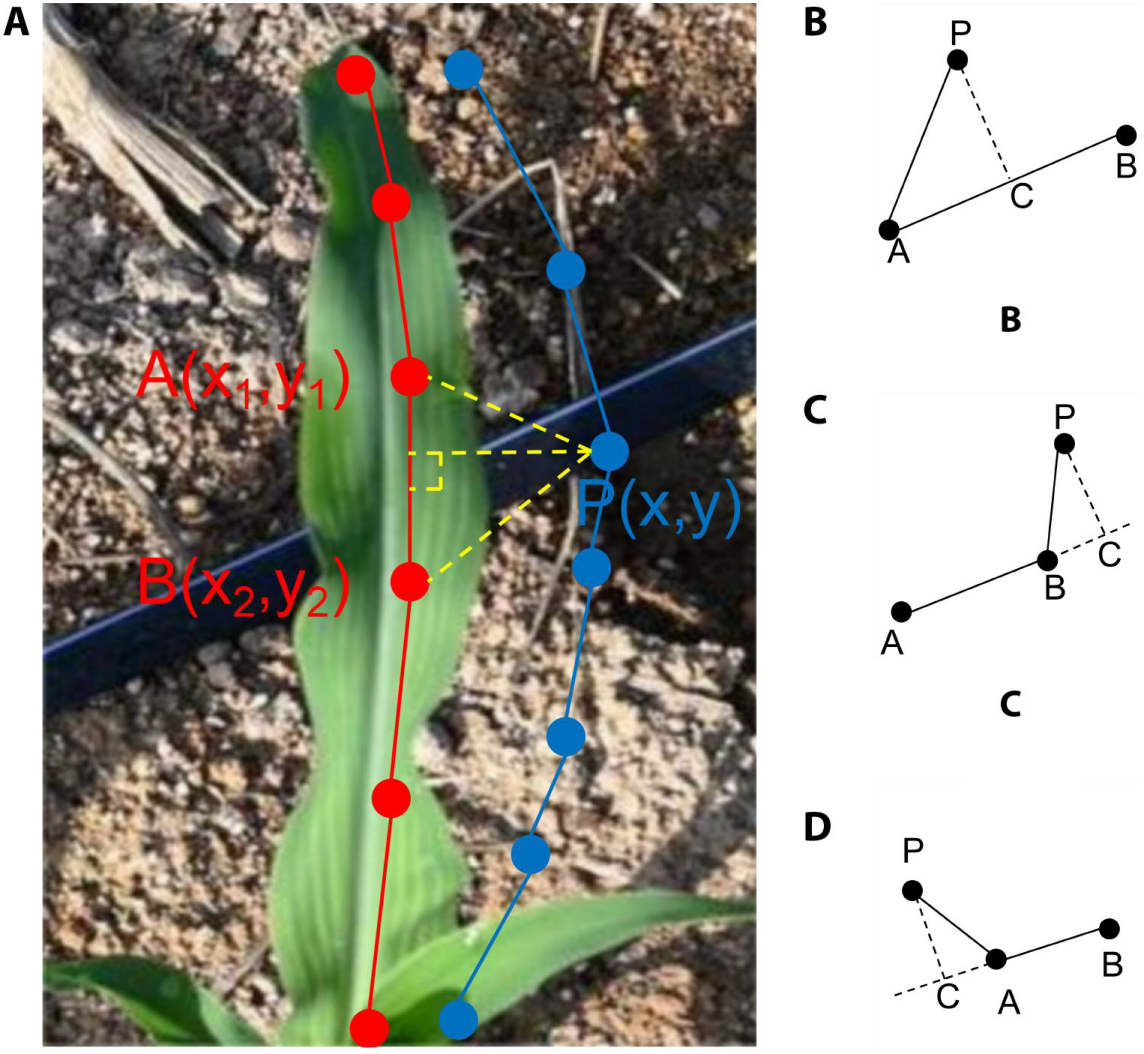
Schematic diagram of the mLD calculation flow: (A) definition of the mLD; (B) the shortest distance from point P to line segment AB is the length of PC; (C) the shortest distance from point P to line segment AB is the length of PB; and (D) the shortest distance from point P to line segment AB is the length of PA.

In detail, $D_{\left(x,y,x_{1}y_{1},x_{2},y_{2}\right)}$ represents the shortest distance from the predicted keypoint (*x*, *y*) to the line segment [(*x*_1_, *y*_1_), (*x*_2_, *y*_2_)], as shown in Fig. 5, and the calculation of the pendant exists in 3 different situations: (b), (c), and (d). $P_{\left(x,y,x_{1}y_{1},x_{2},y_{2}\right)}$ is the coordinate of the pendant foot of the predicted keypoint (*x*, *y*) on the line segment [(*x*_1_, *y*_1_), (*x*_2_, *y*_2_)]; $L_{\left(x,y,x_{1}y_{1},x_{2},y_{2}\right)}$ represents the Euclidean distance between the 2 points (*x*_1_, *y*_1_) and (*x*_2_, *y*_2_); and $C_{\left(x,y,x_{1}y_{1},x_{2},y_{2}\right)}$ represents the inner product of the vector $\vec{\mathrm{AP}}$ and the vector $\vec{\mathrm{AB}}$, as shown in Fig. 5A. The coordinates of A, B, and P are (*x*_1_, *y*_1_), (*x*_2_, *y*_2_), and (*x*, *y*), respectively.

## Results and Discussion

### Experimental parameter settings

The training and validation experiments were performed at the Agricultural Genomics Institute of the Chinese Academy of Agricultural Sciences utilizing a computing server equipped with an NVIDIA Tesla V100S PCle GPU with 32 GB of memory. The operating system used was Linux 3.10.0. For deep learning training and validation, PyTorch 1.10.1 and Cuda 11.1, which are specifically tailored to the server environment, were utilized. As explained in the “Dataset processing” section, the input images were resized from 6,016 × 4,016 to 1,504 × 1,004. Prior to converting the RGB images to tensor format, random color adjustment and random horizontal flipping were applied to enhance the data diversity and improve the model robustness. These preprocessing operations, as demonstrated in previous extensive experiments [45,53,54], have proven to be effective in mitigating overfitting. During the training process, we utilized the stochastic gradient descent optimizer with momentum. The model was trained for 200 epochs, commencing with an initial learning rate of 1e−3 and a weight decay of 1e−4. To reduce the learning rate, we employed the StepLR strategy, which involved decreasing the learning rate by a factor of 0.66 for every 10 fixed steps.

### Results

#### Object detection results for maize based on 3 different original models

In our experiments, we evaluated the object detection accuracy for the maize dataset using 3 popular target detection models: Faster R-CNN, RetinaNet [55], and YOLOv3 [56].

**Fig. 6.**
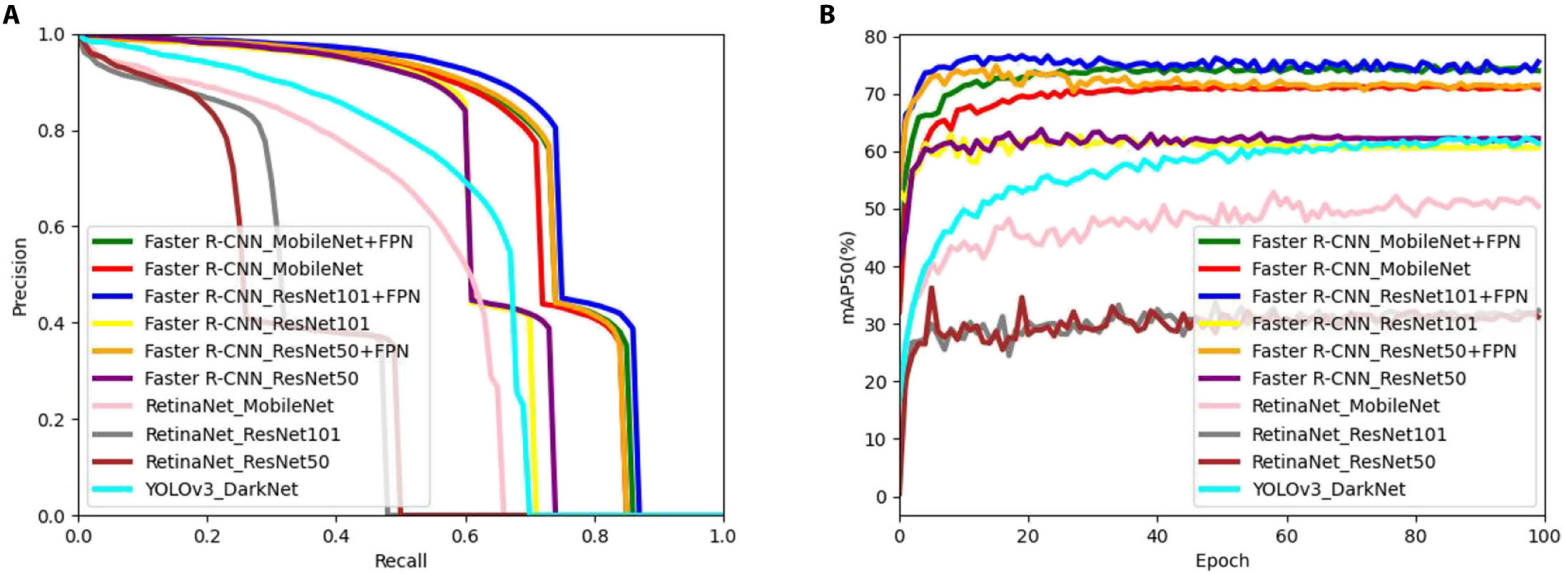
Performance of different object detection models: (A) Precision–recall with different models; (B) mAP50 (%) achieved using different models.

All 3 models can incorporate additional branches for tasks such as keypoint detection or instance segmentation. We did not attempt to modify the default backbone architecture of YOLOv3. While it is technically feasible to replace the backbone with other models such as ResNet50, the original Darknet architecture is well suited for YOLOv3 and generally recommended for optimal performance. We selected the model that achieved the highest accuracy for further study. The object detection results are presented in Fig. 6 and Table 1.

**Table 1.** Object detection results for models with different backbones

Model	Backbone	AP50 (%)		mAP50 (%)	mAP75 (%)	Time (ms)
		Leaf	Stalk			
RetinaNet	MobileNetV2	52.7	49.7	51.2	14.4	45.4
	ResNet50	41.4	22.4	31.9	12.1	87.3
	ResNet101	39.3	28.0	33.7	12.7	113.1
YOLOv3	Darknet	58.2	58.3	58.3	14.6	19.1
Faster R-CNN(default)	MobileNetV2	66.8	78.6	72.7	31.3	34.0
	ResNet50	57.8	68.8	63.3	30.7	51.3
	ResNet101	58.3	65.8	62.1	32.7	74.7
	MobileNetV2+FPN	68.2	80.2	74.2	33.6	32.0
	ResNet50+FPN	68.6	79.0	73.8	35.0	76.1
	ResNet101+FPN	70.4	81.9	76.2	39.9	89.6

Faster R-CNN (default) with ResNet101 + FPN achieved superior performance compared to the other models, with the highest mAP50 of 76.2% and the highest mAP75 of 39.9%. The detection speed, which refers to the time required to detect each image, was measured at 89.6 ms. Although this speed is longer than that of other backbones, it remains within a reasonable range that meets our expectations. It is worth noting that no optimization operations were performed for the results shown in Table 1, and all hyperparameters were employed as described in the original paper. Therefore, we can consider the selection of the model with the best object detection performance for subsequent studies to be reliable.

#### Object detection results for maize based on the improved faster R-CNN

After selecting Faster R-CNN with ResNet101 + FPN as our object detector, we proceeded to improve the accuracy of object detection. As explained in the “Maize detection” section, the accuracy of downstream branches, such as keypoint detection, is greatly influenced by the performance of the detector in top-down object detection models, including Faster R-CNN. First, we fine-tuned certain hyperparameters and conducted corresponding experiments. The results of these experiments are summarized in Table 2.

**Table 2.** Object detection results for Faster R-CNN with fine-tuned hyperparameters

Parameter	Value	AP50 (%)		mAP50 (%)	mAP75 (%)
		Leaf	Stalk		
RPN PRE NMS+RPN POST NMS	2,000 + 1,000	70.4	81.9	76.2	39.9
	3,000 + 1,500	71.0	82.3	76.7	37.6
	4,000 + 2,000	73.1	82.8	78.0	39.8
	5,000 + 2,500	71.7	83.1	77.4	39.1
	8,000 + 4,000	72.8	80.2	76.5	38.3

The results demonstrate that the model performed optimally when the number of retained proposals in the RPN before and after NMS processing was set to 4,000 and 2,000, respectively. Additionally, we made further adjustments as outlined in the “Adjustment of hyperparameters” section. Apart from the aforementioned hyperparameters, we discovered that other factors, such as the aspect ratios of the anchor generator and the IoU thresholds used in NMS processing in the RPN, had minimal impact on the detection accuracy during our experiments. Hence, we followed the settings specified in the original paper for these parameters. To address the issue of target occlusion present in the dataset, we incorporated the Soft-NMS and D IoU techniques. The experimental results are presented in Table 3.

**Table 3.** Object detection results for Faster R-CNN with Soft-NMS and D IoU

NMS	Soft-NMS	D IoU	AP50 (%)		mAP50 (%)	mAP75 (%)
			Leaf	Stalk		
✓			73.1	82.8	78.0	39.8
	✓		75.1	85.4	80.2	46.3
		✓	74.9	84.4	79.7	43.0
	✓	✓	75.5	86.0	80.8	49.2

The results obtained from the abovementioned experiments are in line with our initial hypothesis. Considering the significant occurrence of maize leaf occlusion in our dataset, particularly during the later stages of growth, as depicted in Fig. 1, many targets are closely positioned or occluded. Consequently, when performing NMS processing, the model may mistakenly filter out genuine targets. However, with the incorporation of Soft-NMS and D IoU techniques, these ground-truth targets are retained to a greater extent, leading to an improvement of 2.8% in the mAP50 and 9.4% in the mAP75 of the model.

#### Trajectory recognition results with Point-Line Net

**Fig. 7.**
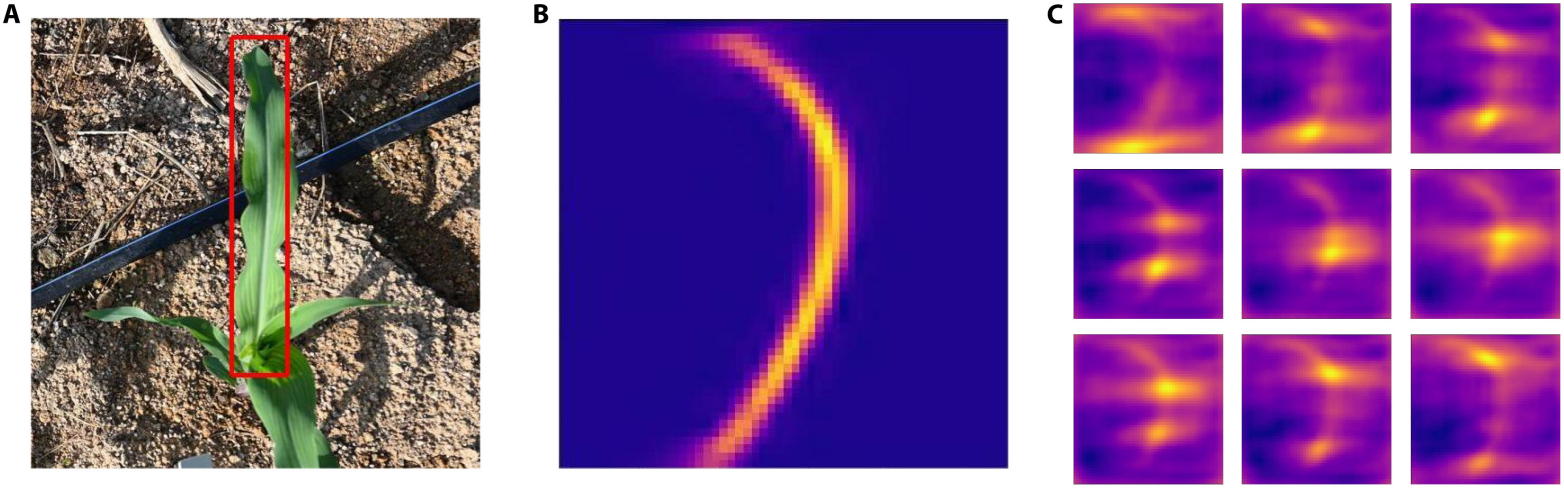
Examples of heatmaps generated by both methods: (A) the selected target instance in the example; (B) a single heatmap inferred by Point-Line Net; and (C) heatmaps inferred by the traditional heatmap-based keypoint detection method.

Inspired by human keypoint detection, we initially attempted to utilize traditional keypoint detection methods to identify the leaf and stalk trajectories of maize. As the experiment progressed, we made the innovative improvements described in the “Modification of the keypoint detection branch” section. We use the inferred heatmaps of a single target to illustrate the difference between the improved method and the traditional keypoint detection method, as well as the superiority of our innovative approach. An example of the comparison is shown in Fig. 7.

**Fig. 8.**
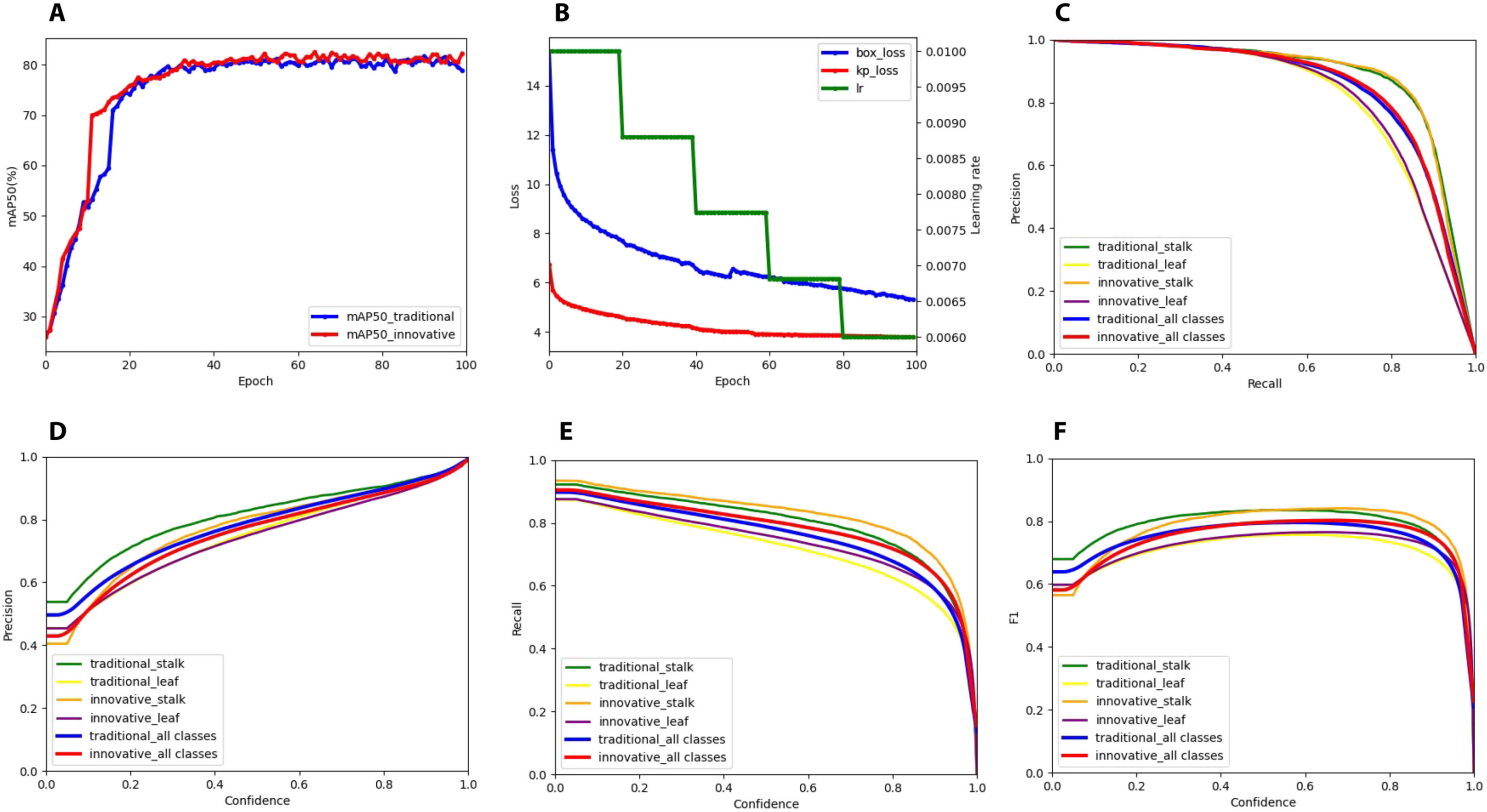
Training and validation performance curves: (A) mAP50 (%) curve changes of traditional and innovative methods; (B) loss curve and learning rate curve changes of traditional and innovative methods, where the box loss consists of RPN loss and Fast R-CNN loss [39], and kp loss refers to the cross-entropy loss of the keypoint detection branch; (C) precision–recall; (D) precision–confidence; (E) recall–confidence; and (F) F1–confidence.

As described in the “Point-Line Net-based Mask R-CNN” section, our improved method discards the traditional heatmap-based keypoint detection approach of inference of one heatmap for each predefined keypoint taking the 9 keypoints as an example, as shown in Fig. 7C, and instead lightweights the inference of just one heatmap for each target instance, which has the advantage of taking into account the fact that during data labeling, these keypoints do not have precise spatial coordinate requirements and as long as they are located on the trajectory of the leaf vein, they are considered to be correctly labeled, unlike the human body keypoint detection task where each keypoint has special location requirements. Because these keypoints have similar feature information, it is easier for the improved network to learn the characteristics of keypoints on the trajectory of the leaf vein. Subsequently, only one NMS operation is required for each target instance to infer the final keypoint coordinates. In contrast, as shown in Fig. 7C, the traditional keypoint detection method requires multiple NMS operations for each target instance, and the heatmap of each keypoint often has multiple peaks, which makes it difficult to perform the correct postprocessing operation. The results of the comparative experiments for the 2 “keypoint” detection methods are presented in Fig. 8 and Table 4:

**Table 4.** Leaf and stalk trajectory detection results using different methods

Method	AP50 (%)		mAP50 (%)	mAP75 (%)	mLD
	Leaf	Stalk			
Traditional method	75.5	86.0	80.8	49.2	34.1
Innovative method	76.5	86.6	81.5	50.1	33.5

The experimental results indicate that our innovative method achieves a smaller distance (measured using custom mLD) between the predicted keypoints of the leaf or stalk and the ground-truth trajectories. Specifically, the distance is reduced to 33.5. This observation further demonstrates that the improvements we have implemented provide better performance in accurately describing the position of the leaf and stalk trajectories according to our requirements. The magnitude of this metric is related to the scale of the input image.

#### Training performance record with Point-Line Net

Throughout the training and validation process, we carefully adjusted the parameters and monitored the performance of the model to identify signs of overfitting or underfitting. We recorded the changes in the learning rate (lr), various losses, and evaluation metrics, as depicted in Fig. 5. The graph illustrates that as the model was trained up to the 100th epoch, the mAP tended to stabilize and would not increase significantly.

**Fig. 9.**
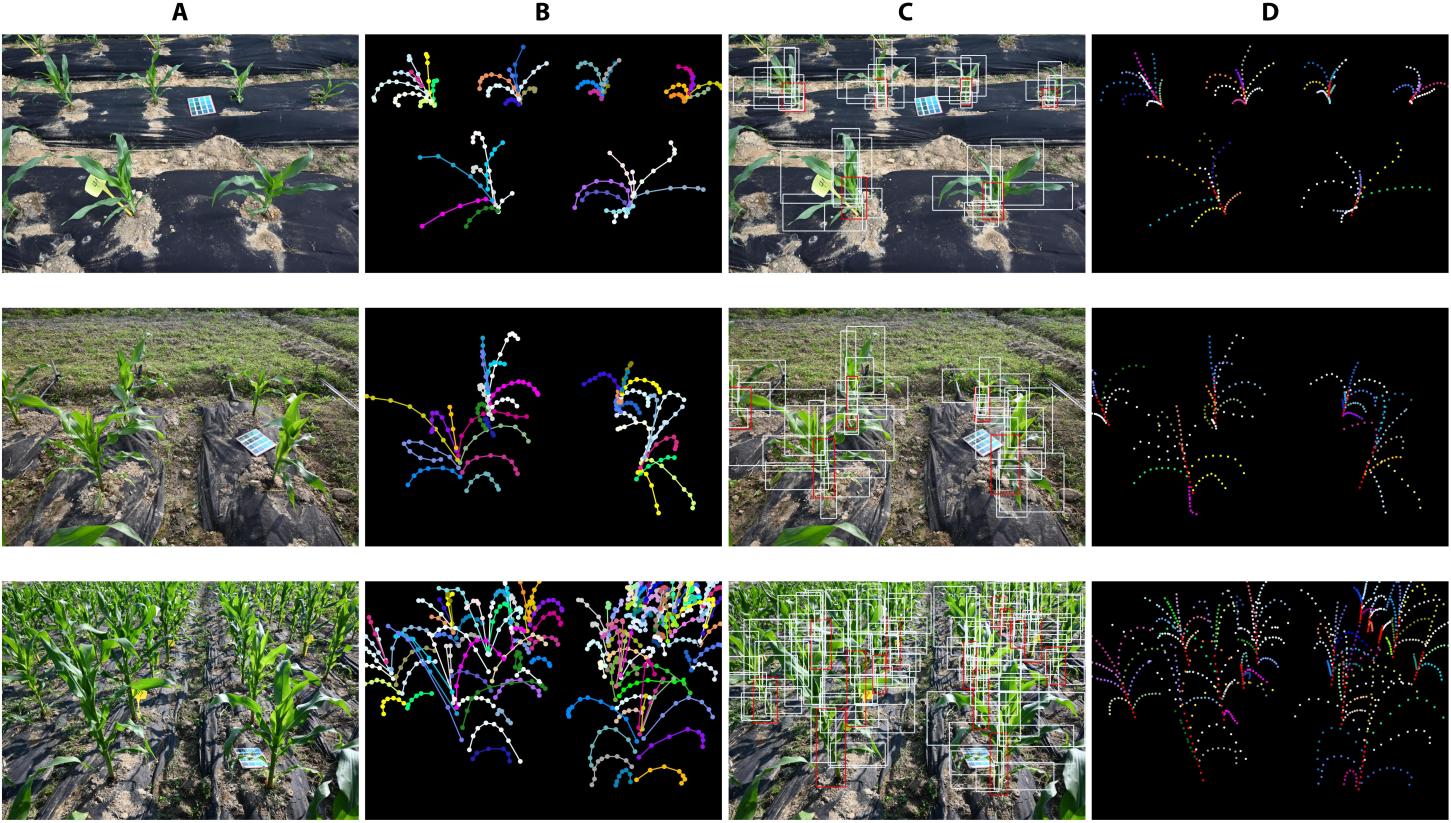
Prediction results of Point-Line Net in various scenarios: (A) original RGB images; (B) ground truth transformed by the Labelme tool; (C) predicted bounding boxes (the red box represents the identified stem, and the white box represents the identified leaf); (D) predicted keypoints.

Although the total loss continued to decrease gradually, there was evidence of overfitting. Consequently, we saved the best weights obtained after the 100th epoch as the final result of model training for subsequent prediction tasks. To showcase the performance of our model, we selected several images from the validation set that presented varying levels of complexity across different growth stages. We then compared the predicted results with the ground-truth annotations. The visualization results are presented in Fig. 9. Notably, as the complexity of the background environment increases and occlusion between targets becomes more prevalent, the tasks of object detection and keypoint detection become progressively more challenging. Nonetheless, our model exhibited commendable performance in such scenarios.

## Conclusion 

Deep learning methods have gained popularity in the field of plant phenotyping due to their exceptional capability for feature extraction and classification [57]. Plant phenotyping aims to quantify various traits of plants, including the size, shape, color, texture, and growth rates of leaves, stems, and flowers. These traits serve as crucial indicators of plant growth, development, and stress responses [58]. They can further be used to facilitate the selection and breeding of superior crops, enhance crop management practices, and predict crop yields. Deep learning methods excel in automatically learning complex and high-dimensional features from plant images, eliminating the need for time-consuming and subjective manual feature engineering. Among these methods, CNNs are particularly renowned for their ability to learn spatial features across multiple scales and orientations from raw images [59].

Since the inception of our maize dataset, our team has been dedicated to utilizing deep learning methods for field crop phenotype detection. To the best of our knowledge, there is currently no field crop phenotypic dataset that employs our innovative dotted-line annotation approach to represent objects, such as leaves or stems, nor is there a dataset as large as ours. Nevertheless, this innovative annotation method, coupled with the complex field environment and the random growth pattern of maize, presents great challenges in achieving accurate detection. Prior to implementing the method proposed in this paper, we attempted various approaches to detect maize leaves using our maize dataset, including instance segmentation. However, none of these methods yielded satisfactory results until we connected this task with human pose estimation, known as multiperson keypoint detection. As the original annotation information lacked ground-truth bounding boxes, we naturally experimented with the bottom-up keypoint detection method described in the “Point-Line Net-based Mask R-CNN” section. Unfortunately, the performance was still inadequate. We assume that handling the clustering problem for multiple targets in complex scenarios, particularly those with significant overlap, proved to be challenging. Based on our previous explorations, we decided to adopt a top-down keypoint detection scheme that incorporated the use of bounding boxes to meet more accurate detection requirements. Subsequently, we fine-tuned the parameters, selected the optimal backbone, and introduced numerous innovative improvements. Each optimization was accompanied by rigorous experimental testing. Ultimately, we achieved an mAP50 of 81.5%, an mAP75 of 50.1%, and a custom distance evaluation index (mLD) of 33.5, which holds great implications for field maize phenotype detection.

Upon reviewing the work presented in this paper, we acknowledge that there is still room for improvement. First, our proposed method is limited by the use of bounding boxes, contrary to the annotation approach employed by our team to create the maize dataset. Consequently, the accuracy of the final keypoint detection is constrained by the accuracy of the initial stage object detection, as mentioned in the “Point-Line Net-based Mask R-CNN” section. In future research, we aspire to achieve the task of detecting leaf and stalk trajectories without the aid of bounding boxes. At the same time, our model has a partial ability to identify the occluded blade, but it does not completely solve the occlusion problem. This is a serious challenge, but subsequent models based on improvements to this dataset, such as adding annotations for the presence or absence of occlusion, may have the ability to address the occlusion challenge. Second, since there is currently no standard evaluation metric for assessing the accuracy of predicted keypoints, we devised the customized index LD. However, there is room for refinement to enhance the comprehensiveness and precision of the evaluation. Additionally, it is important to highlight that our study exclusively employed field images captured by cameras and a fraction of the annotation information provided in our maize dataset. Finally, we did not segment the training for a specific time window during the growth and development of maize, which means that we did not adequately consider the different stress factors or physiological changes that maize may encounter at different growth stages. Moving forward, we aim to conduct more in-depth exploration by incorporating other valuable annotation information, such as specific time windows, leaf attribution information, multiangle location information, and additional images captured by UAVs.

## Data Availability

The computer code and data that support the findings of this study are deposited in a GitHub repository at https://github.com/VEGETALOADING/Point-Line-Net.
